# Nitric oxide and ROS mediate autophagy and regulate *Alternaria alternata* toxin-induced cell death in tobacco BY-2 cells

**DOI:** 10.1038/s41598-019-45470-y

**Published:** 2019-06-20

**Authors:** Abhishek Sadhu, Yuji Moriyasu, Krishnendu Acharya, Maumita Bandyopadhyay

**Affiliations:** 1 Plant Molecular Cytogenetics Laboratory, Centre of Advanced Study, Department of Botany, University of Calcutta, 35, Ballygunge Circular Road, Kolkata, 700019 West Bengal India; 2 Graduate School of Science and Engineering, Saitama University, Shimo-Okubo 255, Saitama, 338-8570 Japan; 3 Molecular and Applied Mycology and Plant Pathology Laboratory, Centre of Advanced Study, Department of Botany, University of Calcutta, 35, Ballygunge Circular Road, Kolkata, 700019 West Bengal India

**Keywords:** Plant cell death, Apoptosis

## Abstract

Synergistic interaction of nitric oxide (NO) and reactive oxygen species (ROS) is essential to initiate cell death mechanisms in plants. Though autophagy is salient in either restricting or promoting hypersensitivity response (HR)-related cell death, the crosstalk between the reactive intermediates and autophagy during hypersensitivity response is paradoxical. In this investigation, the consequences of *Alternaria alternata* toxin (AaT) in tobacco BY-2 cells were examined. At 3 h, AaT perturbed intracellular ROS homeostasis, altered antioxidant enzyme activities, triggered mitochondrial depolarization and induced autophagy. Suppression of autophagy by 3-Methyladenine caused a decline in cell viability in AaT treated cells, which indicated the vital role of autophagy in cell survival. After 24 h, AaT facilitated Ca^2+^ influx with an accumulation of reactive oxidant intermediates and NO, to manifest necrotic cell death. Inhibition of NO accumulation by 2-(4-Carboxyphenyl)-4,4,5,5-tetramethylimidazoline-1-oxyl-3-oxide (cPTIO) decreased the level of necrotic cell death, and induced autophagy, which suggests NO accumulation represses autophagy and facilitates necrotic cell death at 24 h. Application of N-acetyl-L-cysteine at 3 h, confirmed ROS to be the key initiator of autophagy, and together with cPTIO for 24 h, revealed the combined effects of NO and ROS is required for necrotic HR cell death.

## Introduction

Through the course of their lifespan, plants encounter numerous pathogenic invasions, and thus, mount multilayered intrinsic programs to combat such attacks. Such programs monitor and discern a myriad of external and internal stimuli and when considered appropriate, steer the cell either to clear long-lived proteins and worn-out organelles or to initiate an altruistic suicidal cascade for successful existence of the plant. In the host plant, recognition of a pathogen-encoded protein or pathogen-associated molecular patterns (PAMPs), by a host surveillance system (resistant [R] protein), is often associated with rapid, localised cell death, known as hypersensitivity response (HR)^1,2^. Studies on diverse host-pathogen combinations revealed the compelling correlation between the phenomena of oxidative stress, nitrosative burst and host response^3–5^. The accumulation of reactive oxygen intermediates (ROI), e.g. apoplastic generation of superoxide (${{\rm{O}}}_{2}^{\cdot -}$) ions, or its dismutation product hydrogen peroxide (H_2_O_2_)^6–8^ and nitric oxide (NO), respectively are the early events of HR^9–11^. Interestingly, no NOS-like enzymes are present in higher land plants but such enzymes were found in several algal species^12,13^. Several lines of literature suggest in land plants NO is synthesized predominantly by nitrate reductase (NR), from polyamines and hydroxylamines, and via other non-enzymatic routes^14–18^. An oxidative burst followed by HR is a successful host defence policy against biotrophic pathogens^5^, whereas the role of mycotoxin-induced reactive species upon necrotrophic manifestation is still under investigation. Howlett^19^ proposed that necrotrophic fungi manipulate an array of toxins and subvert the host defence process of programmed cell death (PCD), to derive nutrition from dead host tissues.

The mechanism of PCD differs between plant and animal kingdoms^20,21^. Substantially, two major types of cell death mechanisms have been hypothesised to be associated with HR: vacuolar or autophagic cell death as a plant innate immune response^22^, and necrotic cell death as a cell suicidal reaction^23^. Loss of cytoplasmic extent with a significant increase in the volume occupied by lytic vacuoles, invagination and fusion of vacuolar membranes with vesicles for subsequent cargo degradation and eventually tonoplast rupture, and discharge of vacuolar hydrolases can be assigned as autophagic cell death markers^21^. On the other hand, necrosis is identified by lipid degradation and plasma membrane damage, loss of mitochondrial activity, shrinkage of the protoplast and unprocessed remains of cell fragments^24^. Studies on *Nicotiana benthamiana* and *Arabidopsis* plants with silenced or knocked-out *A*u*T*opha*G*y (*ATG*) genes have changed the perception of autophagy during HR. Initially characterised as a cytoprotective cellular manoeuvre during pathogen intrusion, autophagy takes part in limiting the spread of HR symptoms and disease associated cell death response following viral and fungal infection^10,25–29^.

The ubiquitous presence of the pathogenic fungus *Alternaria alternata* (Fr.) Keissler causes a serious worldwide depletion of economic yield^30^. In *Nicotiana tabacum* (tobacco), the pathogen has been reported to inculcate lethal symptoms like anthracnose, black root rot, frog eye leaf spot, verticillium wilt and brown spots. Among these diseases, brown spot predominantly engenders more than 50 per cent depletion in global tobacco production^31^. The pathogenesis of *A*. *alternata* is primarily toxin-mediated^32,33^. The resilience of these necrotrophs in the injection of host-selective or non-host-selective toxins (HSTs or NHSTs) (e.g., tenuazonic acid (TeA), alternariol (AOH), alternariol monomethyl ether (AME), brefeldin A, tentoxin, zinniol)^34^ within the host tissue, are keys for successful disease manifestation. The cytotoxic *A*. *alternata* extract^35^ further purified to obtain crude toxin^36^, activated caspase-like proteases and induced reactive oxygen species (ROS) but no DNA fragmentation (the hallmark feature of apoptosis). Contrary to this observation Cheng *et al*.^37^ reported *A*. *alternata* metabolic extract-induced apoptosis-like PCD in tobacco BY-2 cells. However, a thorough exploration of *A*. *alternata* toxin (AaT)-induced disruption of cellular homoeostasis and cell death as a consequence of HR is absent.

Assessment of the effects of elicitors *in planta* is rather cumbersome, as the manifestation of toxic effects often initiates in unreachable small groups of cells concealed by surrounding healthy cells^38^. In contrast, cells in suspension being less complex and with enhanced sensitivity towards external stressors, render the ease of the analysis. In our previous work, we had provided evidence and suggested that AaT facilitated NO generation, and induced defence enzyme activity and phenolics accumulation in *Rauvolfia serpentina* callus^39^. In this study, we report a thorough evaluation of AaT-incited intracellular consequences in terms of altered calcium ion (Ca^2+^) concentration, accumulation of ROS and reactive nitrogen species (RNS), evaluation of redox balance in terms of reduced and oxidized glutathione ratio (GSH/GSSG), mitochondrial depolarization, antioxidant profile, autophagy and toxin-induced cell death, in cultured wild-type (wt) and transgenic BY-2 cells expressing GFP-Atg8 protein. We further assessed the occurrence of AaT-induced autophagy simultaneously, in the presence of NO scavenger 2-(4-Carboxyphenyl)-4,4,5,5-tetramethylimidazoline-1-oxyl-3-oxide (cPTIO), autophagic phosphatidylinositol 3-kinase (PI3K) inhibitor 3-methyladenine (3-MA) and ROS scavenger N-acetyl-L-cysteine (NAC). Our results substantiate autophagy to be a pro-survival signal during HR and an active NO-dependent regulation of autophagy. Additionally, NO-mediated inhibition of autophagy triggers necrotic cell death. However, repression of NO by cPTIO, keeps the autophagic cascade switched on during prolonged exposure to the necrotrophic toxin.

## Results

### AaT spikes intracellular ROS and NO generation in congruence with Ca^2+^ accumulation

**Figure 1:**
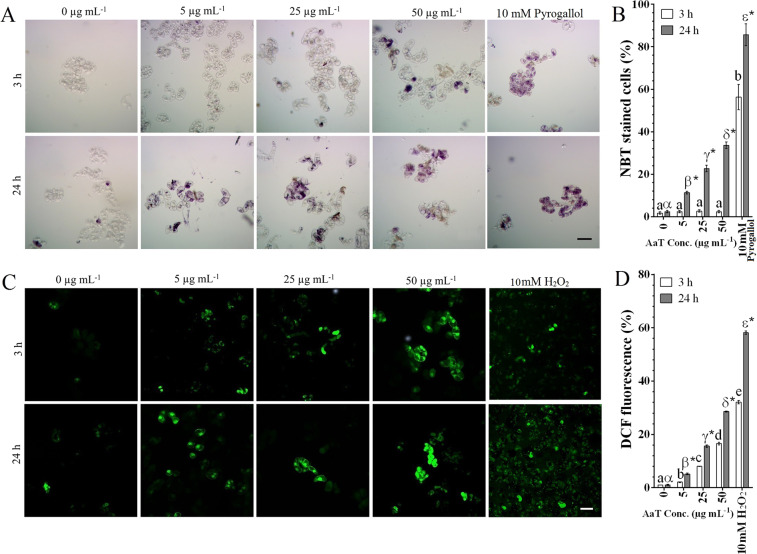
*Alternaria alternata* toxin-induced accumulation of ROS in BY-2 cells treated for 3 and 24 h. Histochemical visualization of (**A**) ${{\rm{O}}}_{2}^{\cdot -}$ generation by NBT staining and (**B**) graphical representation of the same. (**C**) Observation of ·OH, ROO· and H_2_O_2_ accumulation by the fluorescent probe DCFH-DA. (**D**) Spectroflurimetric estimation of DCF fluorescence. Scale bars denote 50 µm. Different Roman letters (3 h) or Greek letters (24 h) represent significant differences (*P* < 0.05) compared to control by Holm–Sidak multiple comparison test. Asterisks (*) depict the significant difference (*P* < 0.05) at same AaT concentration at different time points.

**Figure 2:**
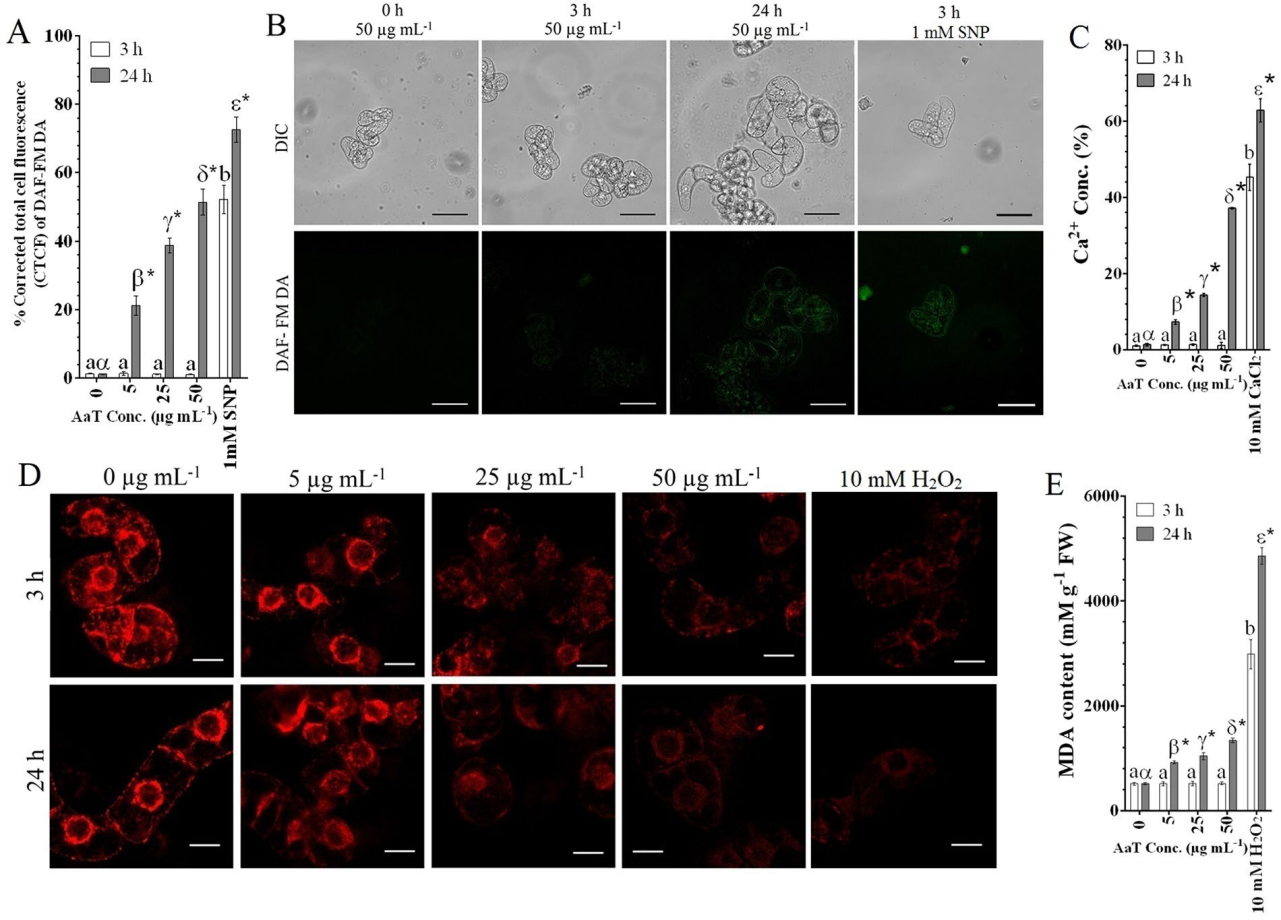
NO generation and estimation of Ca^2+^ influx induced by *Alternaria alternata* toxin at 3 and 24 h in BY-2 cells and consecutive effects on mitochondria and membranes. (**A**) Quantification of DAF-FM DA fluorescence by ImageJ software. (**B**) Fluorescent photomicrographs of DAF-FM DA stained BY-2 cells treated with 50 µg mL^−1^ AaT [Scale bars denote 50 µm]. (**C**) Analysis of intracellular Ca^2+^ upsurge in tobacco cells. (**D**) Loss of mitochondrial membrane potential represented by quenching of Rhodamine 123 fluorescence [Scale bars denote 20 µm]. (**E**) ROS induced membrane lipid peroxidation represented by increased MDA content. Different Roman letters (3 h) or Greek letters (24 h) represent significant differences (*P* < 0.05) compared to control by Holm–Sidak multiple comparison test. Asterisks (*) depict the significant difference (*P* < 0.05) at same AaT concentration at different time points.

Previously^39^, we had determined the optimum concentration of AaT for the promotion of pathogenicity in *R*. *serpentina* callus to be 50 µg mL^−1^. To extend our observations, we assessed the immediate (after 3 h) and prolonged (after 24 h) aftermath of AaT application in tobacco BY-2 cells. NBT staining of AaT-treated cells revealed a notable accumulation of ${{\rm{O}}}_{2}^{\cdot -}$ only after 24 h (Fig. 1A,B): ~33.7% of cells treated with 50 µg mL^−1^ of AaT exhibited blue formazan precipitation. Although a few cells seemed to accumulate blue formazan after 3 h at 50 µg mL^−1^, no statistical difference (*P* > 0.05) was detected compared to control. However, upon DCFH-DA staining, AaT exposed cells showed the significant occurrences of hydroxyl (·OH), peroxyl (ROO·) intermediates especially H_2_O_2_ after 3 h, which increased consecutively at 24 h (Fig. 1C,D). DAF-FM DA staining revealed NO accumulation only after 24 h of AaT treatment with the highest accumulation at 50 µg mL^−1^ (Fig. 2A,B). o-CPC method revealed no significant (*P* > 0.05) alteration in Ca^2+^ concentration at 3 h. Nonetheless, a pronounced dose dependant increase in intracellular Ca^2+^ concentration was observed after 24 h of AaT treatment (Fig. 2C), which was detected highest at 50 µg mL^−1^ AaT. Thus at 24 h, increased Ca^2+^ concentration can be correlated with the generation of ${{\rm{O}}}_{2}^{\cdot -}$, the rise in H_2_O_2_ level and NO accumulation.

### AaT-induced ROS causes mitochondrial membrane depolarization and membrane lipid peroxidation

AaT-treated-BY-2 cells showed concentration-dependent loss of mitochondrial membrane potential (Δ*Ψ*_m_), mirrored by a significant decrease (*P* < 0.05) in Rh 123 fluorescence intensities (Fig. 2D). Interestingly, the rapid generation of H_2_O_2_ upon AaT exposure was analogous with the loss of Δ*Ψ*_m_ (Supplementary Fig. S1A). Furthermore, AaT-induced decline in mitochondrial dehydrogenase and oxidoreductase activities affirmed mitochondria to be the target of ROIs, which in turn contributed to the cellular ROS pool [Supplementary Fig. S1B,C]. Though significant ROS induction was measured within 3 h of toxin exposure, no significant alteration in MDA production (lipid peroxidation marker) was noticed (Fig. 2E). However, after 24 h, significant accumulation (*P* < 0.05) of MDA was observed, with the highest production at 50 µg mL^−1^ AaT exposure. This suggests that though detectable alteration of ROS homeostasis initiates post 3 h AaT exposure, the deleterious effect of oxidative stress culminates after 24 h.

### AaT interferes with the cellular antioxidant defence system

**Figure 3:**
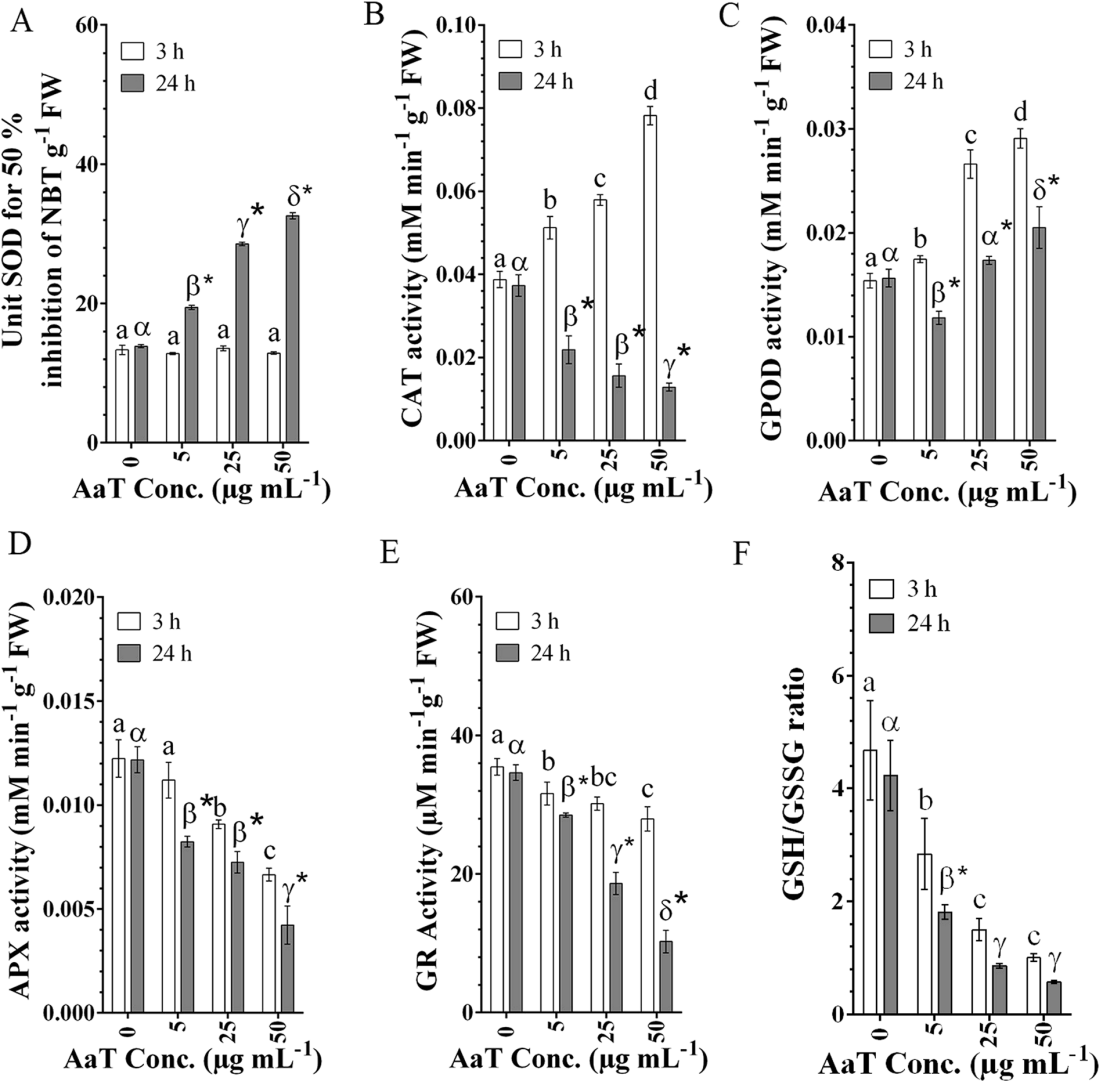
*Alternaria alternata* toxin-induced alterations in enzymatic and non-enzymatic antioxidant activities in tobacco BY-2 cells treated for 3 and 24 h. (**A**) Increase in Superoxide dismutase (SOD) activity at 24 h. (**B**) Increased Catalase (CAT) activity at 3 h, followed by a significant decline at 24 h. (**C**) Enhanced guaiacol peroxidase (GPOD) activity at 3h, followed by decreased activity after 24 h compared to that of at 3 h. (**D**) Dose-dependent decline in Ascorbate peroxidase (APX) activity. (**E**) Dose-dependent decline in Glutathione reductase (GR) activity. (**F**) The decrease in reduced and oxidized glutathione (GSH/GSSG) ratio. Different Roman letters (3 h) or Greek letters (24 h) represent significant differences (*P* < 0.05) compared to control by Holm–Sidak multiple comparison test. Asterisks (*) depict the significant difference (*P* < 0.05) at same AaT concentration at different time points.

Alterations in key regulators of ROS scavenging networks were examined upon AaT exposure, in congruence with the increment in ROS accumulation. SOD activity did not alter after 3 h of toxin treatment, which emulated the observations made in NBT staining, i.e., inconsequential ${{\rm{O}}}_{2}^{\cdot -}$ generation incapable of inciting SOD activity (Fig. 3A). Conversely, after 24 h, toxin-treated cells showed a concomitant dose-dependent increase in ${{\rm{O}}}_{2}^{\cdot -}$ generation as well as SOD activity. Treated cells showed ~2.5 fold increment in SOD activity than control cells at the highest toxin concentration. A gradual increase in CAT activity was noted in congruence with the generation of H_2_O_2_ at 3 h (Fig. 3B). Interestingly, at prolonged incubation for 24 h, CAT activity decreased in a concentration-dependent manner. GPOD activity spiked ~1.13, ~1.72 and ~1.88 fold with increasing toxin concentrations, respectively, in accordance with the ROS level (Fig. 3C). However, after 24 h, GPOD activity declined significantly (*P* < 0.05) compared to that after 3h.

Activities of the two terminal enzymes of ascorbate-glutathione (ASC-GSH) cycle, APX and GR showed a dose-dependent decline; though the decrease in activities was greater between 3 to 24 h than that from 0 h to 3 h (Fig. 3D,E). A consequent loss in GSH/GSSG ratio was also recorded (Fig. 3F) which suggests increasing oxidative stress upon toxin treatment.

### AaT induces necrotic cell death in BY-2 cells

**Figure 4:**
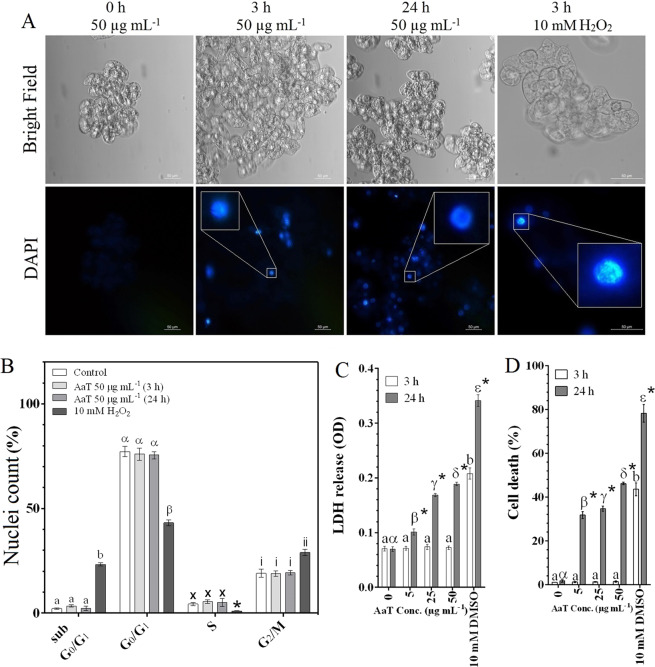
Assessment of cell death in *Alternaria alternata* toxin treated tobacco BY-2 cells with positive control 10 mM H_2_O_2_. (**A**) Fluorescent photomicrographs of DAPI stained BY-2 cells treated with 50 µg mL^−1^ AaT at 0, 3 and 24 h. (**B**) Flow cytometric estimation of cell cycle progression and AL-PCD like DNA fragmentation in tobacco cells, at 50 µg mL^−1^ AaT at 0, 3 and 24 h. (**C**) Estimation of necrosis by lactate dehydrogenase (LDH) leakage. (**D**) Spectrophotometric quantification of cell death by Evans blue staining. Scale bars denote 50 µm. Graph bars with the same letters or symbols are statistically similar (*P* < 0.05) according to Holm–Sidak multiple comparison test. Asterisks (*) depict the significant difference (*P* < 0.05) at same AaT concentration at different time points.

Two major types of PCDs, apoptosis-like cell death (AL-PCD) and necrosis, were evaluated to understand the effects of AaT on cell death. Chromatin condensation and DNA fragmentation, distinctive features of the apoptotic nuclei, were assessed by DAPI staining. Though a few cells were stained with DAPI after 3 h of AaT treatment (statistically insignificant), maximum cell death was observed after 24 h; the nuclei observed were normal, maintaining a mesh-like chromatin structure (Fig. 4A). This trend was consistently observed in multiple fields in different independent experiments. In contrast, H_2_O_2_-treated cells displayed the typical hallmarks of apoptotic nuclei, i.e. condensed and fragmented chromatin material. In accordance with the observations from DAPI staining, flow cytometric evaluation suggested lack of DNA fragmentations in BY-2 cells, in response to AaT. DNA fragments lost from apoptotic nuclei were assessed by detecting the broad hypodiploid (sub-G_0_/G_1_) peak, flow cytometrically. Results revealed insignificant differences (*P* > 0.05) among the sub-G_0_/G_1_ population of control and AaT treated cells (50 µg mL^−1^) after 3 h and 24 h (Fig. 4B). However, H_2_O_2_ treated cells showed the highest ~23.7% hypodiploid (sub-G_0_/G_1_) peak [Supplementary Fig. S2**]**. Moreover, a high number of G_2_/M nuclei observed in H_2_O_2_ treated cells supported the cells’ strategy to withstand DNA damage, by either providing time to repair or activate apoptosis-like PCD. Therefore, flow cytometric evaluation also showed the absence of DNA fragments, in AaT treated cells.LDH is a cytoplasmic enzyme that is released from necrotic cells through damaged plasma membrane into the extracellular matrix. LDH release was not detected after 3 h of AaT treatment (Fig. 4C). However, 24 h of AaT treatment caused a significant increase in LDH release, which ascertained membrane damage and necrotic cell death. In congruence with the LDH data, no significant difference (*P* > 0.05) in Evans Blue dye uptake was observed in post 3 h AaT treated cells, whereas, concentration-dependent dye uptake was observed after 24 h (Fig. 4D). All these data supported the hypothesis of necrotic cell death in BY-2 cells.

### NO-mediated inhibition of autophagy initiates necrotic PCD

**Figure 5:**
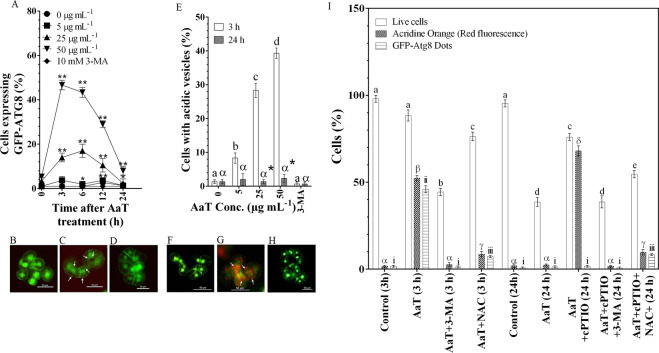
Characterization of *Alternaria alternata* toxin-induced autophagy at 3 and 24 h using tobacco BY-2 cells expressing transgenic GFP-ATG8 protein (**A**–**D**) and by Acridine orange (AO) staining (green fluorescence [533 nm] and red fluorescence [656 nm] merged) (**E**–**H**). (**A**) Percentage of transgenic GFP-ATG8 tobacco BY-2 cells showing the formation of GFP-ATG8 dots as a marker of autophagy. *, ** Indicate differences between control and AaT-treated cells that are significant at the 1 and 5% levels by Holm–Sidak multiple comparison test. (**B**) Control cells, (**C**) 50 µg mL^−1^ AaT treated cells, (**D**) 50 µg mL^−1^ AaT + 10 mM 3-MA-treated GFP-ATG8 BY-2 cells. (**E**) Percentage of AO stained wild-type tobacco BY-2 cells showing acidic vesicles. Different Roman letters (3 h) or Greek letters (24 h) represent significant differences (*P* < 0.05) compared to control by Holm–Sidak multiple comparison test. Asterisks (*) depict the significant difference (*P* < 0.05) at same AaT concentration at different time points. (**F**) Control cells, (**G**) 50 µg mL^−1^ AaT treated cells, (**H**) 50 µg mL^−1^ AaT + 10 mM 3-Methyladenine (3-MA) treated cells stained with AO. (**I**) Evaluation of toxin-induced cell death and autophagy with a different combination of 100 µM NO scavenger cPTIO, 10 mM autophagic inhibitor 3-Methyladenine (3-MA) and 250 µM ROS scavenger N-Acetyl-L-cysteine (NAC). Different Roman letters, Greek letters and Roman numerals represent significant differences (*P* < 0.05) compared to control (3 h) by Holm–Sidak multiple comparison test.

We assessed whether AaT was capable of inducing autophagy using BY-2 cells expressing GFP-Atg8 fusion protein. At 50 µg mL^−1^ of AaT, GFP-Atg8 dots i.e. autophagosomes, increased after 3 h and decreased after 6 h (Fig. 5A–D). Upon AO-staining, we occasionally detected small vesicles that emit red fluorescence, probably lysosomes or autolysosomes, in the cells treated with AaT. The number of cells having such AO-stained small vesicles increased at 3 h after treatment with AaT (Fig. 5E–H). These acidic vesicles were not seen after 24 h. These results show that autophagy was transiently activated around 3 h after treatment with AaT.

**Figure 6:**
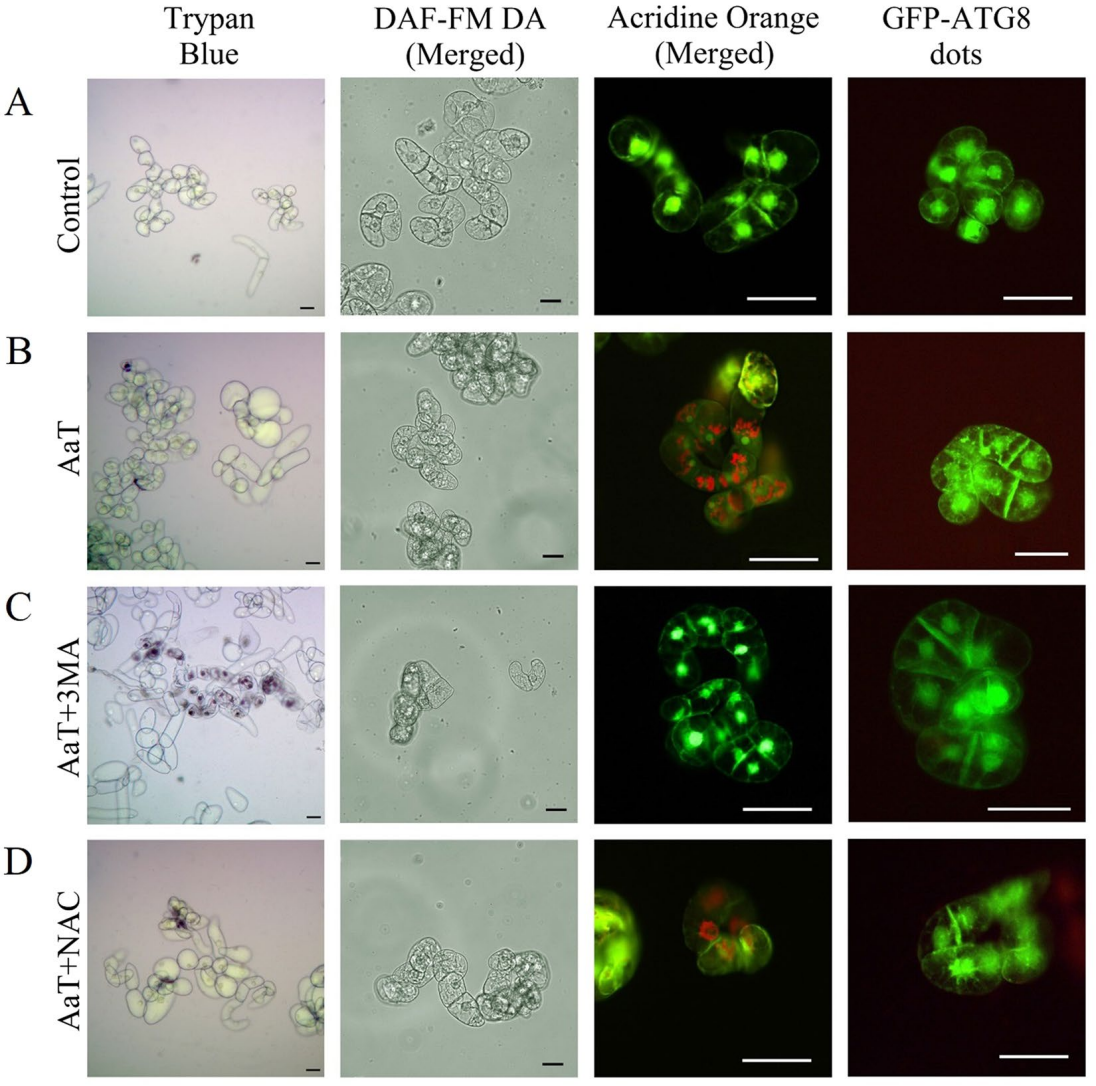
Correlation among cell death, nitric oxide (NO) and autophagy in tobacco BY-2 cells after 3 h of *Alternaria alternata* toxin (AaT) exposure. (**A**) The control wild-type untreated cells, (**B**) 50 µg mL^−1^ AaT, (**C**) 50 µg mL^−1^ AaT + 10 mM 3-MA, (**D**) 50 µg mL^−1^ AaT + 250 µM NAC treated wild-type cells stained with trypan blue, DAF-FM DA, AO and GFP-ATG8 cells. Scale bars denote 50 µm.

**Figure 7:**
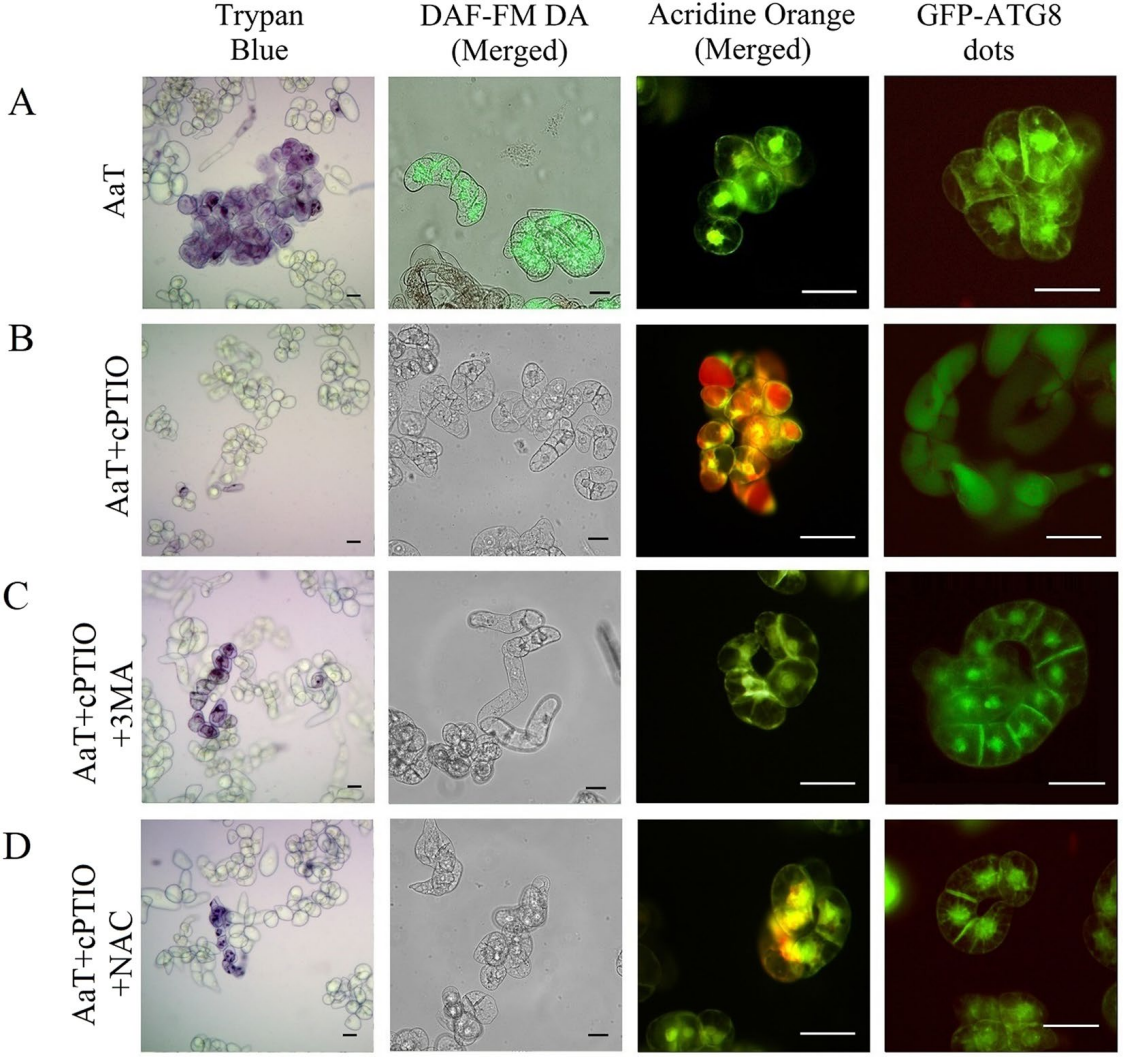
Correlation among cell death, nitric oxide (NO) and autophagy in tobacco BY-2 cells after 24 h of *Alternaria alternata* toxin (AaT) exposure. (**A**) 50 µg mL^−1^ AaT, (**B**) 50 µg mL^−1^ AaT + 100 µM cPTIO, (**C**) 50 µg mL^−1^ AaT + 100 µM cPTIO + 10 mM 3-MA, (**D**) 50 µg mL^−1^ AaT + 100 µM cPTIO + 250 µM NAC treated wild type cells. Scale bars denote 50 µm.

In order to resolve the correlation among autophagy, ROS, and RNS, we used the autophagy inhibitor 3-MA, ROS scavenger NAC, and NO scavenger cPTIO. The autophagy inhibitor 3-MA inhibited autophagy activated 3 h after AaT treatment and lowered cell viability to 50% (Figs 5I and 6C). Thus, autophagy was found to play a role in cell survival upon AaT exposure. Addition of NAC also inhibited autophagy, but ~80% of cells were found to be viable (Fig. 6D). Hence, ROS was likely to be involved in the induction of autophagy as well as in the promotion of cell death. After 24 h of AaT exposure, BY-2 cells showed a reduction in cell viability to 40% with a notable accumulation of NO (Fig. 7A). Inhibition of NO by cPTIO restored cell viability to 80% (Fig. 7B) and showed a significant reduction of LDH release from the AaT + cPTIO (24 h) treated cells (Supplementary Fig. S3**)**. These results suggest that NO potentiated cell death.

Administration of cPTIO also caused interesting morphological changes. AO staining revealed numerous particles, probably cytoplasmic materials, emitting red fluorescence, distributed in the central vacuoles (Fig. 7B). Moreover, GFP-Atg8 cells showed diffused GFP fluorescence throughout the central vacuole, although unlike at 3 h, accumulation of autophagosomes was not seen at 24 h. 3-MA blocked the emergence of AO-stained particles and accumulation of GFP fluorescence in the central vacuoles. Thus these morphological changes evoked by treatment with cPTIO were likely to be the outcome of autophagy (Fig. 7C). Therefore these results showed that inhibition of NO accumulation by cPTIO kept the autophagy mechanism switched on, which in turn interrupted the necrotic cell death.

## Discussion

Innate immune response in plants generally comprises PCD^40^. Though *ATG* genes work in tandem with HR by either restricting or promoting PCD^41^, the manifestation of autophagy during AaT-induced HR response is ambiguous. In the current investigation, we report the onset of autophagy as an initial response to AaT in BY-2 cells. However, upon longer exposure to AaT, NO accumulation triggered necrotic cell death, together with ROS. Moreover, we propose a NO-mediated modulation of autophagy.

ROS accumulation after 3 h post AaT treatment without significant alteration in ${{\rm{O}}}_{2}^{\cdot -}$ generation indicates the direct generation of H_2_O_2_. Various oxidases such as amino-acid oxidase, glucose oxidase, glycolate oxidase, and sulfite oxidase generate H_2_O_2_ by hyper-oxidation of their respective substrates^42^. Therefore, it can be assumed that such oxidases contributed to the accumulation of H_2_O_2_ under AaT stress condition. On the other hand, dysfunction of the mitochondrial dehydrogenases (e.g. succinate dehydrogenase) and oxidoreductases (e.g. NAD(P)H-ubiquinone oxidoreductase) by AaT, jeopardised the electron transport system, which in turn stimulated ROS. Furthermore, Brefeldin A present in AaT^43^ affects the endoplasmic reticulum and Golgi hybrid (Supplementary Fig. S4) which also contributes to ROS upheaval^44,45^.

At 24 h, the concomitant increase in intracellular Ca^2+^ and ${{\rm{O}}}_{2}^{\cdot -}$, supports the NADPH-dependent oxidase complex (NOX)-mediated ROS accumulation. This can be corroborated with the host-pathogen incompatible interaction^8,46,47^. NOX is regulated by Ca^2+^ via direct binding to the EF-hand motifs and phosphorylation by Ca^2+^ dependent protein kinases, which catalyzes the generation of ${{\rm{O}}}_{2}^{\cdot -}$, predominantly in apoplasm^48,49^. The spike in H_2_O_2_ pool, observed in 24 h AaT exposed cells are thus the results of dismutation of the ${{\rm{O}}}_{2}^{\cdot -}$ and subsequent liberation of H_2_O_2_ in various sub-cellular locations. Since NO signaling mechanism of plants is comparable to that of animals^14^, the slow increment of Ca^2+^, which peaked at 24 h, can be corroborated with NO accumulation. However, further studies are required to confirm the actual source of NO generation upon AaT treatment. Therefore, this biphasic ROS accumulation and concomitant generation of NO during incompatible interaction (at 24 h) suggest the onset of HR^50^.

The intracellular ROS and NO levels may influence the antioxidant enzyme activities. At insignificant NO levels, high antioxidant activities were detected with moderate ROS generation. Since most of the antioxidants, with the exception of SOD (dismutation of the increased accumulation of ${{\rm{O}}}_{2}^{\cdot -}$), decreased notably after 24 h compared to that of at 3 h, and significant generation of NO was observed at 24 h, it can be assumed that the presence of NO likely affected the antioxidant activities and promoted ROS accumulation. To keep the intracellular ROS at equilibrium CAT activity along with GPOD peaked after 3 h. However, at 24 h sharp decline of CAT and reduction of GPOD compared to that of at 3 h, can be corroborated with the decrease in cell viability. Moreover, toxin and time-dependent decrease in the GSH/GSSG ratio clearly indicates the oxidative stress induced by AaT. The progressive depletion of APX, GR and GSH at 3 h could be the consolidated effects of ROS and autophagy. Though APX and CAT are two major enzymatic ROS scavengers, APX has a higher affinity toward H_2_O_2_ (in µM range) than CAT (in mM range)^51^. Moreover, APX is ubiquitous in every ROS producing cellular compartment; whereas, CAT is present exclusively in peroxisomes. Therefore, our results suggest that at 3 h, organelles such as mitochondria and plastids which possess the Foyer-Halliwell-Asada cycle (ASC-GSH cycle) were more prone to oxidative damage than peroxisomes as they possess CAT and peroxidases. Furthermore, in mitochondria, ‘ROS-induced ROS release’ establishes a positive-feedback loop that causes augmentation in ROS production and results in perceptible mitochondrial damage^52,53^. Thus, removal of such worn-out organelles via selective autophagic pathway^54^ can be the probable cause of diminution of APX, GR, and GSH at 3 h. The decline in enzymatic antioxidant activity at 24 h, compared to that at 3 h, can be related to the NO accumulation. NO is capable of several post-translational modifications such as nitration and *S*-nitrosylation^55^. Reports suggest that CAT, APX, and GR are the targets of NO in tobacco and arabidopsis^56–60^. Hence, it is likely that at 24 h the augmented NO level of AaT treated cells could also be a cause of low APX and GR activity together with increased cell death. Under nitrosative stress, high GSSG and the *S*-nitrosylation of GSH with NO (produces *S*-Nitrosoglutathione) are likely to decrease the GSH pool^61^. Nevertheless, further studies are required to confirm this hypothesis.

In the present study, AaT-induced mode of cell death is characterised as necrotic cell death after 24 h (Supplementary Fig. S3). Our findings support the hypothesis that continuous ROS generation for several hours stimulates the necrotic cell death pathway^62^. Here, we observed after 24 h, AaT triggered loss of Δ*Ψ*_m_, plasma membrane damage, protoplast shrinkage, accumulation of Ca^2+^, ROS and RNS; however, no DNA fragmentation occurred. All these observations cumulatively affirm that AaT induces HR-PCD in BY-2 cells^50^. Cheng *et al*.^37^ reported *A*. *alternata* metabolic products induced DNA laddering during induction of PCD. However, the metabolic products they used were different from the “crude toxin” used in this study. Moreover, in congruence with our study, Yakimova *et al*.^63^ validated true ‘crude toxin’ does not initiate DNA fragmentation in tobacco leaves. Víteček *et al*.^64^ also reported that NO- and H_2_O_2_-induced PCD does not accompany DNA fragmentation generally observed during AL-PCD. Furthermore, several lines of literature support that plants lack the ‘classic’ apoptotic regulatory network and canonical caspases, and the caspase-like activity noticed during HR and disease incited PCD, is due to protease activity, especially, vacuolar processing enzymes^21,65^.

The manifestation of autophagy at 3 h validates autophagy-mediated cell survival response against *A*. *alternata* by BY-2 cells, and thus inhibition of autophagy by 3-MA in AaT treated cells (3 h), promoted cell death. On the other hand, at 3 h, NAC inhibited the accumulation of ROS, as well as the onset of autophagy, which suggests that the ROS-induced by AaT facilitated the induction of autophagy. It has been shown that the external application of H_2_O_2_ and methyl viologen incited macroautophagy in *Arabidopsis*^66^. Unlike 3-MA, NAC however, did not promote cell death, which indicates ROS to be the cardinal executors of cell death. Therefore, it can be inferred that at 3 h, AaT-triggered ROS, instigated the pro-survival role of autophagy, and inhibition of autophagy led to ROS-induced cell death.

The addition of cPTIO along with AaT for 24 h inhibited the accumulation of NO, interrupted HR-PCD and instigated autophagy. These observations support that NO is an essential messenger in cell death execution during HR^67^. In mammalian cells, it has been reported that autophagy is suppressed by NO via PtdIns3K complex Vps34/Beclin1^68,69^. Though several *ATG* genes are known to be associated with HR either by regulating the spread of cell death symptoms^10,25,26^ or by commencing HR at the site of infection^22^, the correlation between NO and autophagy in plants was unclear. To the best of our knowledge, we report for the first time that prevention of NO accumulation triggers the onset of autophagy during HR.

The occurrence of bright red vesicles upon AO staining suggested that autophagy was activated by cPTIO exposure in AaT treated cells at 24 h. However, in contrast to the AaT treated cells at 3 h, AO staining of AaT and cPTIO (24 h) treated cells showed accumulation of cytoplasmic materials in the central vacuoles. Activation of autophagy was further confirmed by the increase in diffused GFP fluorescence in the central vacuoles. The occurrence of cytoplasmic materials and GFP in the vacuolar lumen suggests the fusion of autolysosomes with the vacuole, i.e. the final phase of the autophagic mechanism. Treatment of AaT and cPTIO cells with 3-MA for 24 h showed loss of cell viability as well as inhibition of autophagy. Therefore, it can be assumed that the inhibition of NO by cPTIO re-initiated autophagy, while 3-MA further repressed autophagy which consequently led to cell death. Addition of NAC validated that ROS was responsible for decreased cell viability in 3-MA-treated cells.

Taken together, our findings substantiate the *modus operandi* of disease manifestation by *A*. *alternata*. Results of the present study revealed the unknown correlations among ROS, NO and autophagy during HR response. Initially, upon necrotrophic assault, the upsurge in ROIs (·OH, ROO·, H_2_O_2_) activates the onset of autophagy as a pro-survival defence strategy. However, prolonged AaT exposure triggers the Ca^2+^ signaling cascade which amplifies oxidative stress and facilitates NO generation to manifest cell death. Furthermore, inhibition of NO accumulation provided evidence for the occurrence of autophagic response. Thus, results affirm NO to be a possible regulator of cell survival and/or cell death during HR, apart from ROS. Therefore, our study established the rationale behind the success of *A*. *alternata* for widespread crop failure and opens up new avenues of research using various host-pathogen and/or HST/NHST combinations to discover novel roles of rudimental cellular mechanisms.

## Materials and Methods

### AaT production and purification

Four weeks old *A*. *alternata* mycelia grown on PDA were inoculated in Richard’s Medium to obtain culture filtrate for toxin purification. The toxin was obtained as crude toxin, following Slavov *et al*.^36^ with modifications^39^.

### Cell line and toxin treatment

Tobacco BY-2 cells were grown and subcultured in MS medium as previously described^70^. Log-phase cells (4 days after subculture) were exposed to AaT, at varying concentrations (0, 5, 25 and 50 µg mL^−1^) based on our previous work^39^. Cells were treated with the toxin in MS medium with 50 mg mL^−1^ cell concentration at 25 ± 2 °C in 25 mL Erlenmeyer flasks (separately for each concentration and time point) under agitation (120 rpm) in darkness to assess its immediate (after 3 h) and sustained (after 24 h) response.

### Intracellular ROI detection

Intracellular production of ${{\rm{O}}}_{2}^{\cdot -}$ and H_2_O_2_ were assessed using nitro blue tetrazolium (NBT) and 2′, 7′-Dichlorofluorescin Diacetate (DCFH-DA) staining described by Santos *et al*.^71^ and Huang *et al*.^72^ respectively. 10 mM pyrogallol and 10 mM hydrogen peroxide (H_2_O_2_) treatments were used as positive control. NBT stained samples were observed under a bright field microscope (BFM) (Carl Zeiss, Primostar) and photographed using AxioCamERc5s Camera (Carl Zeiss). The percentage of NBT stained cells were counted from 300 cells from three independent experiments (100 × 3) and converted to percentage. For H_2_O_2_ detection, toxin-treated cells, 1 mL of treated cells (50 mg mL^−1^) from each concentration were taken in PBS were stained with DCFH-DA at 5 µM final concentration. After 1 h of incubation at 25 ± 1 °C in dark, cells were washed thoroughly with PBS. Cells were studied by a confocal laser scanning microscope (CLSM; Olympus IX81 microscope, Olympus, Japan; λ_ex_ = 488 and λ_em_ = 520 nm). Photomicrographs were acquired using Olympus FLUOVIEW software (Ver. 04.02.02.09; Olympus, Japan), and the fluorescent intensities of 50 mg treated cells, were detected using a fluorescence spectrophotometer (Hitachi F-7000, Japan; λ_ex_ = 488 and λ_em_ = 520 nm). DCF fluorescence intensity was measured is expressed as percentage fold change of relative fluorescent unit (r.f.u.) over control (at 3 h), considering control fluorescence as 1%.

### 4-Amino-5-Methylamino-2′, 7′-Difluorofluorescein Diacetate (DAF-FM DA) staining of AaT induced NO

Post 3 h and 24 h, estimation of NO generation in 0.5 mL of AaT treated cells (50 mg mL^−1^) from each concentration, were detected following Gupta *et al*.^39^. 1mM sodium nitroprusside (SNP) treated cells were used as positive control. For NO specific fluorescent staining, 1 mL of treated cells (50 mg mL^−1^) from each concentration cells were stained with 10 µM of DAF-FM DA in PBS for 15 min in dark at RT. Photomicrographs were acquired by CLSM (λ_ex_ = 495 and λ_em_ = 515 nm). The intensity of DAF-FM DA fluorescence was quantified from 50 cells from three independent experiments (50 × 3) by Image J software and calculated in terms of corrected total cell fluorescence (CTCF) as follows:

$${\rm{CTCF}}={\rm{Integrated}}\,{\rm{density}}-({\rm{Area}}\,{\rm{of}}\,{\rm{selection}}\times {\rm{mean}}\,{\rm{background}}\,{\rm{fluorescence}})$$

### Analysis of AaT-induced alteration in Calcium ion (Ca^2+^) concentration

The change in total Ca^2+^ concentration in AaT treated cells were assessed using the o-cresolphthalein complexone (o-CPC) method^73,74^ due to its high specificity towards Ca^2+^. Since MS medium contain CaCl_2_ (~3 mM), toxin treated cells were washed thoroughly with PBS to remove any trace of culture medium. 10 mM CaCl_2_ treated cells were used as positive control. Total Ca^2+^ concentrations from 25 mg toxin treated cell homogenates were detected in a reaction mix containing 0.375 M Ethanolamine (pH 10.6), 82 µM o-CPC, 7.16 mM 8-Hydroxyquinoline, 27.75 mM HCl. The Ca-o-CPC complex was bichromatically measured at 570/660 nm in 96-well plate, the resulting increase in absorbance of the reaction mixture being directly proportional to the Ca^2+^ concentration in the lysate, and is expressed in terms of percentage fold change over control (at 3 h), considering control OD as 1%.

### Detection of ROS-induced loss of mitochondrial membrane potential (Δ*Ψ*_m_) and Lipid peroxidation (LPO)

AaT-induced mitochondrial vitiation in terms of alteration of Δ*Ψ*_m_ was analysed using Rhodamine 123 (Rh 123) fluorescent probe^72^. 1 mL of treated cells (50 mg mL^−1^) from each concentration were stained with 2.5 µM Rh 123 at 25 °C for 30 min. After washing rigorously, fluorescent photomicrographs were obtained using CLSM (λ_ex_ = 507 and λ_em_ = 529 nm). LPO was estimated using thiobarbituric acid reactive substances (TBARS) assay measuring malondialdehyde (MDA) according to Ghosh *et al*.^75^. Absorbance was measured at 532 nm spectrophotometrically and corrected for non-specific turbidity by subtracting the absorbance at 600 nm. MDA content was estimated using the extinction coefficient of thiobarbituric acid reactants (TBAR) (ε = 155 mM cm^−1^) and expressed as mM g^−1^ fresh weight (FW). 10 mM H_2_O_2_ treated cells were used as the positive control for both the experiments.

### Determination of intracellular enzymatic antioxidant activity

#### Protein extraction

After incubation, cells were collected from 6 mL AaT treated suspension by centrifugation at 300 × *g* for 1 min. The pelleted cells were washed and homogenised with 1 mL of 10 mM TRIS-HCl (pH 8; tris (hydroxymethyl) aminomethane), 1 mM EDTA (ethylenediaminetetraacetic acid), 0.5 mM EGTA (ethylene glycol- bis (2-aminoethylether)-N,N,N′,N′-tetraacetic acid), 140 mM NaCl (sodium chloride), 1 mM PMSF (phenylmethylsulfonyl fluoride), 1% Triton X- 100 and 1.5% PVP (Polyvinylpyrrolidone). For 15 min at 4 °C, the cell homogenates were centrifuged at 12000 × *g*. Bradford method^76^ was used to measure the soluble protein content in the supernatant. BSA (bovine serum albumin) was used as the standard.

Enzymatic antioxidants, viz. superoxide dismutase (SOD; EC 1. 15. 1. 1), catalase (CAT; EC 1. 11. 1. 6), guaiacol peroxidase (GPOD; EC 1. 11. 1. 7), ascorbate peroxidase (APX; EC 1. 11. 1. 11), and glutathione reductase (GR; EC 1.6.4.2) were estimated by the following methods.

#### Spectrophotometric assay of enzymes

SOD activities of AaT exposed cells were estimated following Beauchamp and Fridovich^77^ and Achary *et al*.^78^. The absorbance of formazan so formed was taken at 560 nm, and expressed as unit SOD g^−1^ FW (1 unit of SOD activity = amount of the enzyme that causes 50% inhibition of NBT reduction).CAT activity was studied following Aebi^79^. The decline in absorbance due to H_2_O_2_ (ε = 39.4 mM^−1^ cm^−1^) degradation was estimated at 240 nm for 2 min and expressed in mM min^−1^ g^−1^ FW. Following Chance and Maehly^80^, peroxidase activity was estimated, based on oxidation of guaiacol (ε = 26.6 mM^−1^ cm^−1^) to tetra-guaiacol. The rise in absorbance for 1 min at 470 nm was shown in mM min^−1^ g^−1^ FW. Following Gallego *et al*.^81^, APX activity was measured immediately in fresh extracts. The enzyme activity was measured for 2 min at 265 nm as a decrease in the absorbance and expressed in mM min^−1^ g^−1^ FW. Following Smith *et al*.^82^, GR activity was assayed by the increase in absorbance at 412 nm due to glutathione-dependent reduction of Ellman′s Reagent [5, 5′- Dithiobis (2- nitrobenzoic acid), DTNB] to 2-nitro- 5-thiobenzoic acid (TNB) (ε = 14.15 M^−1^ cm^−1^) and the unit was expressed as nM min^−1^ g^−1^ FW.

#### Determination of GSH/GSSG ratio

The redox balance in the toxin-treated tobacco cells were estimated by determining the GSH: GSSG ratio spectrophotometrically following Anderson^83^ with modifications^84^ at 412 nm. Treated cells were homogenized in 6% metaphosphoric acid containing 1 M EDTA, followed by centrifugation at 12000×*g* for 15 min at 4 °C.

The supernatant was divided into two parts to measure total glutathione and GSSG respectively. Total glutathione was estimated in a reaction mixture containing potassium phosphate buffer (pH 7.5), DTNB, BSA and NADH. The mixtures were incubated at 37 °C for 15 min. The change in absorbances were detected at 412 nm and expressed as µM g^−1^ FW. For the estimation of GSSG, 2-vinylpyridine was added to the supernatant to remove GSH for 1 h at 25 °C. The sample extract further added to a reaction buffer (phosphate buffer containing EDTA, pH 7.5), diluted yeast glutathione reductase (GR) and DTNB. The reaction was initiated by addition of NADPH. The change in absorbance was measured at 412 nm and expressed as µM g^−1^ FW. GSH content was determined after subtracting GSSG from the total glutathione. GSH and GSSG was calculated from 1 mM stock diluted in 6% metaphosphoric acid.

#### Detection of apoptotic nuclei using 4′,6-Diamidine-2′-phenylindole dihydrochloride (DAPI)

For precise qualitative determination of the effect of AaT-induced PCD, BY-2 nuclei were stained using DAPI at the final concentration of 10 µg mL^−1^ at RT^85^. The cells were studied under Leica DM IL LED (Leica, Wetzlar, Germany) fluorescent microscope. Images were taken by Leica DFC 450C camera (Leica, Wetzlar, Germany) using Leica Application Suite V.4.7.1 software (Leica, Wetzlar, Germany) using a blue filter. At least 500 cells were observed in each of the three replicate slides per sampling time per treatment from three independent experiments.

#### Analysis of apoptosis by propidium iodide staining and flow cytometry

1 mL of AaT treated BY-2 cells (50 mg mL^−1^), after incubation in 1% cellulase for 1 h, was frozen and chopped to isolate the nuclei. Following Riccardi and Nicoletti^86^, isolated nuclei stained with propidium iodide (PI) were sieved through 50 µm nylon mesh and analysed in BD FACSVerse™ Flow Cytometer. 1200 nuclei were analysed at medium flow rate (60 µL min^−1^), to detect the broad hypoploid (sub-G_0_/G_1_) peak corresponding to apoptotic cell population. The nuclei count under each population were gated and expressed as percentage nuclei count, obtained from the machine statistics.

#### Lactate dehydrogenase (LDH) activity

The magnitude of necrotic cell death was measured based on membrane permeabilization and release of the cytoplasmic enzyme, LDH, from the damaged cells. The released LDH was measured from 0.5 mL of treated cells (50 mg mL^−1^) from each concentration, based on its ability to form coloured formazan from 2-piodophenyl-3-p-nitrophenyl-5-phenyl tetrazolium chloride (INT)^87^. 10 mM dimethyl sulfoxide (DMSO) treated tobacco cells were used as positive control. The change in absorbance was detected at 490 nm and expressed as an increase in OD, which is directly proportionate to the LDH release from cells, i.e. necrotic cell death.

#### Evans Blue cell death assay

0.5 mL of treated cells (50 mg mL^−1^) from each concentration were analysed following Ohno *et al*.^88^ at 3 h and 24 h time points. Absorbed dye was extracted in 50% methanol with 1% SDS for 1 h at 60° C, quantified spectrophotometrically at 600 nm, and expressed as percentage fold change over control (at 3h) considering control dye absorption as 1%. 10 mM DMSO treated cells were used as positive control.

#### Identification of autophagic vesicles by Acridine orange (AO)

Visualisation of intracellular acidic compartments was carried out by staining the cells with basic fluorescent dye AO^89^. Treated cells, washed in PBS were stained with 20 µM of AO at 25 °C in dark for 30 min. Cells were washed thoroughly in PBS at least thrice, to wash off any extra unbound stain and observed under fluorescent microscope (FM; Leica DM IL LED; Leica, Wetzlar, Germany). Photomicrographs were taken by Leica DFC 450C camera (Leica, Wetzlar, Germany) and Leica Application Suite V.4.7.1 software (Leica, Wetzlar, Germany) using green (λ_ex_ = 490 and λ_em_ = 525 nm) and red (λ_ex_ = 532 and λ_em_ = 650 nm) fluorescence filter. Cells with acidic vesicles were counted from 300 cells from three independent experiments (100 × 3) and expressed as percentage.

#### Cytoplasmic localisation of the GFP-ATG8 fusion protein

The toxin-induced onset of autophagy was investigated in transformed tobacco cells expressing GFP-Atg8 fusion protein^90^. BY-2/GFP-ATG8 cells cultured in MS medium like its non-transformed counterpart was treated with the aforementioned toxin concentrations and scanned under FM for cytosolic GFP-ATG8 dot formation. Cells showing the onset of autophagy were counted from 300 cells from three independent experiments (100 × 3), and expressed as percentage. Fluorescent photomicrographs were acquired by FM using GFP filter.

#### Treatment with NAC, cPTIO and 3-MA

To elucidate the correlation between AaT and autophagy, wt and GFP-Atg8 BY-2 cells were subjected to AaT (50 µg mL^−1^) along with different combinations of 250 µM NAC, 100 µM cPTIO and 10 mM 3-MA. In brief, cells were treated in (a) AaT alone, (b) AaT + 3-MA, (c) AaT + NAC for 3 h and (d) AaT, (e) AaT + cPTIO, (f) AaT + cPTIO + 3-MA and (g) AaT + cPTIO + NAC for 24 h and cells without any treatment was used as control. Subsequently, the wt cells were stained using trypan blue and observed under BFM; AO, DAF-FM DA stained wt cells, and GFP-Atg8 cells were analysed under FM. A total of 300 cells from three independent experiments (100 × 3) were counted and expressed as percentage.

### Statistical analyses

Each assay was performed at least thrice with three replicas. Statistical analyses were done in SigmaPlot 12.1 software. Values are shown in the graphs as the Mean ± Standard deviation (SD). Changes in absorbance and frequencies of cell counts are expressed as fold change over control (3 h). Two- and one-way analysis of variance (ANOVA) were done to establish statistical correlations among obtained data. When ANOVA showed significant difference Holm–Sidak’s *post hoc* test was applied at 1%, and 5% (different lower-case letters) probability level considering time and AaT concentrations as main factors for pairwise comparison and AaT concentrations as the main factor for comparison with a control group.

### Supplementary information

Supplementary Figures S1-S4
