# Author Correction: Nitric oxide and ROS mediate autophagy and regulate *Alternaria alternata* toxin-induced cell death in tobacco BY-2 cells

**DOI:** 10.1038/s41598-024-55895-9

**Published:** 2024-03-04

**Authors:** Abhishek Sadhu, Yuji Moriyasu, Krishnendu Acharya, Maumita Bandyopadhyay

**Affiliations:** 1 Plant Molecular Cytogenetics Laboratory, Centre of Advanced Study, Department of Botany, University of Calcutta, 35, Ballygunge Circular Road, Kolkata, 700019 West Bengal India; 2 Graduate School of Science and Engineering, Saitama University, Shimo-Okubo 255, Saitama, 338-8570 Japan; 3 Molecular and Applied Mycology and Plant Pathology Laboratory, Centre of Advanced Study, Department of Botany, University of Calcutta, 35, Ballygunge Circular Road, Kolkata, 700019 West Bengal India

Correction to: *Scientific Reports* 10.1038/s41598-019-45470-y, published online 20 June 2019

This Article contains errors.

Due to mistakes during figure assembly, Figure 6A “DAF-FM DA” control is a partial duplication of Figure 7B “DAF-FM DA” Aat+cPTIO.

The corrected Figure 6 and its accompanying legend appear below.

**Figure 6:**
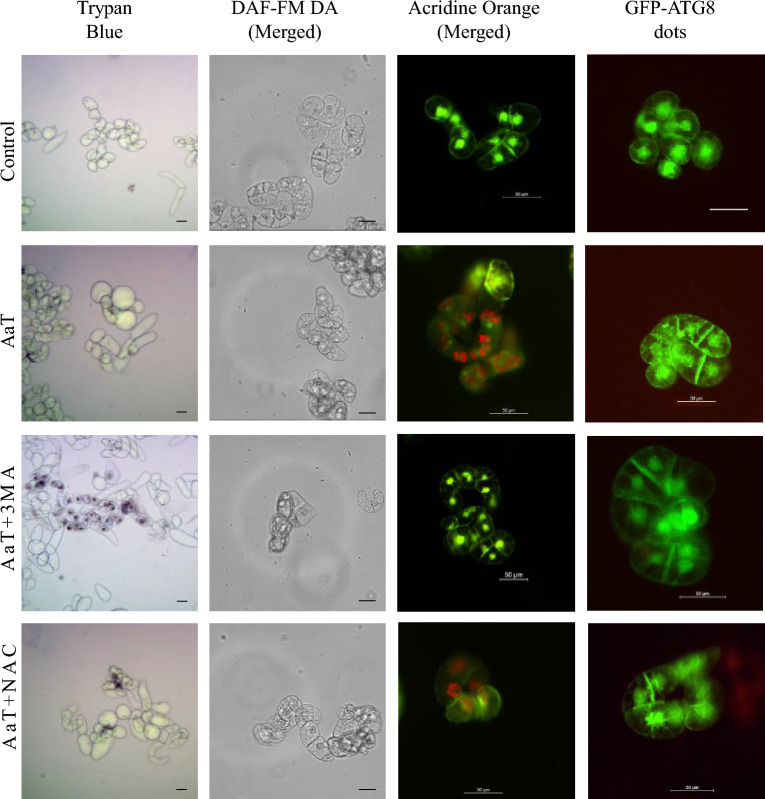
Correlation among cell death, nitric oxide (NO) and autophagy in tobacco BY-2 cells after 3 h of *Alternaria alternata* toxin (AaT) exposure. (**A**) The control wild-type untreated cells, (**B**) 50 µg mL^−1^ AaT, (**C**) 50 µg mL^−1^ AaT + 10 mM 3-MA, (**D**) 50 µg mL^−1^ AaT + 250 µM NAC treated wild-type cells stained with trypan blue, DAF-FM DA, AO and GFP-ATG8 cells. Scale bars denote 50 µm.

Additionally, Figure 5B and D are identical to Figure 6A and C “GFP-ATG8 dots” control and AaT+3MA, respectively, and Figure 5F and H are identical to Figure 6A and C “Acridine Orange (Merged)” control and AaT+3MA, respectively. These panels represent the same condition.

