# Supplementary material for: Nitric oxide and ROS mediate autophagy and regulate Alternaria alternata toxin-induced cell death in tobacco BY-2 cells
Source: Sci Rep. 2019 Jun 20;9:8973. doi: 10.1038/s41598-019-45470-y (PMC6586778; doi:10.1038/s41598-019-45470-y)
Supplement: Supplementary file 1 — Supplementary Figures S1-S4 [file 41598_2019_45470_MOESM1_ESM.pdf]

Title: Nitric oxide and ROS mediate autophagy and regulate *Alternaria alternata* toxin-induced cell death in tobacco BY-2 cells

Authors: Abhishek Sadhu, Yuji Moriyasu, Krishnendu Acharya, Maumita Bandyopadhyay

Supplementary Figure S1

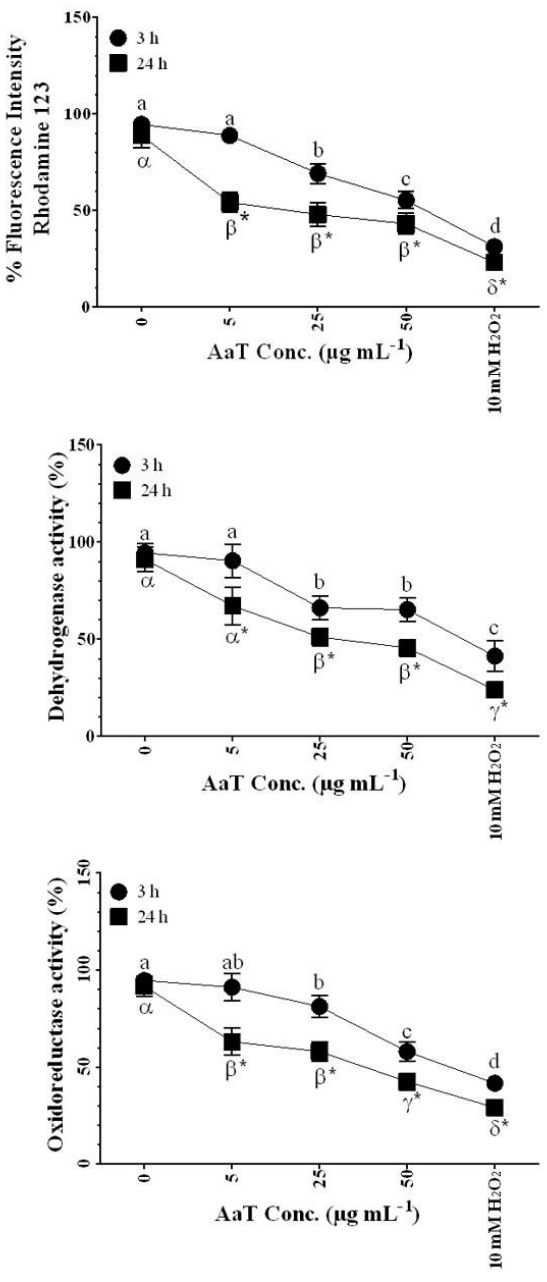

Fig. S1. Effect of AaT on BY-2 mitochondria. (A) Percentage decrease of Rh 123 fluorescence depicting loss of membrane polarization (fluorescent intensities of 50 mg treated cells, were detected using a fluorescence spectrophotometer [Hitachi F-7000, Japan; λex = 507 and λem = 529 nm]. Rh 123 fluorescence intensity was measured and is expressed in terms of percentage fold change of relative fluorescent unit [r.f.u.] over control [at 3 h]). Mitochondrial dehydrogenase and oxidoreductase activity of 0.5 mL of AaT treated cells were analyzed independently in separate assays, analyzing the reduction of 1- (4, 5-dimethylthiazol-2-yl)- 3, 5- diphenyltetrazolium bromide (MTT) and 1, 3, 5- triphenyl tetrazolium chloride (TTC) to formazan. After incubating the cells with tetrazolium salts following Babula *et al*.[1] and Towill and Mazur[2], formazan was extracted in their respective solubilizing solutions. Absorbance was read on iMark™ Microplate Absorbance Reader (BIO-RAD, USA) at 570 nm, with 630 nm (as a reference wavelength) (MTT) and at 490 nm (TTC), after loading into 96-well flat bottom plates. Each activity is expressed in terms of percentage fold change over the activity of control tobacco cells (at 3 h). (B) Loss of mitochondrial dehydrogenase activity detected using MTT. (C) Loss of mitochondrial oxidoreductase activity detected using TTC. Different Roman letters (3 h) or Greek letters (24 h) represent significant differences (P<0.05) compared to control by Holm–Sidak multiple comparison test. Asterisks (*) depict the significant difference (P<0.05) at same AaT concentration at different time points.

Supplementary Figure S2

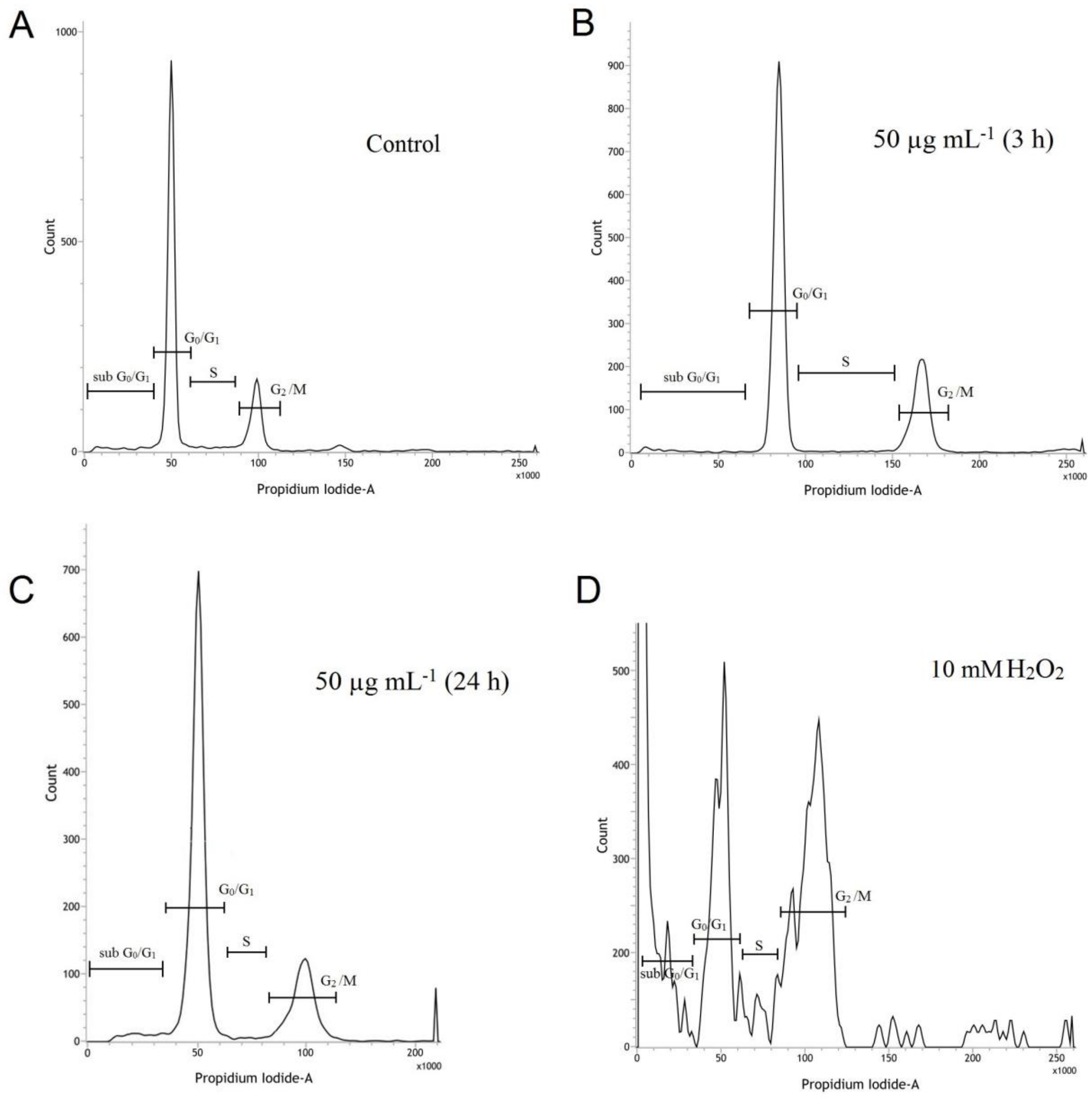

Fig. S2. Representative FCM histograms of AaT treated BY-2 cells. (A) Control BY-2 cells (3 h), (B) 50 µg mL^{-1} AaT treated BY-2 cells for 3 h, (C) 50 µg mL^{-1} AaT treated BY-2 cells for 24 h, (D) 10 mM H_2O_2 treated BY-2 cells used as positive control.

Supplementary Figure S3

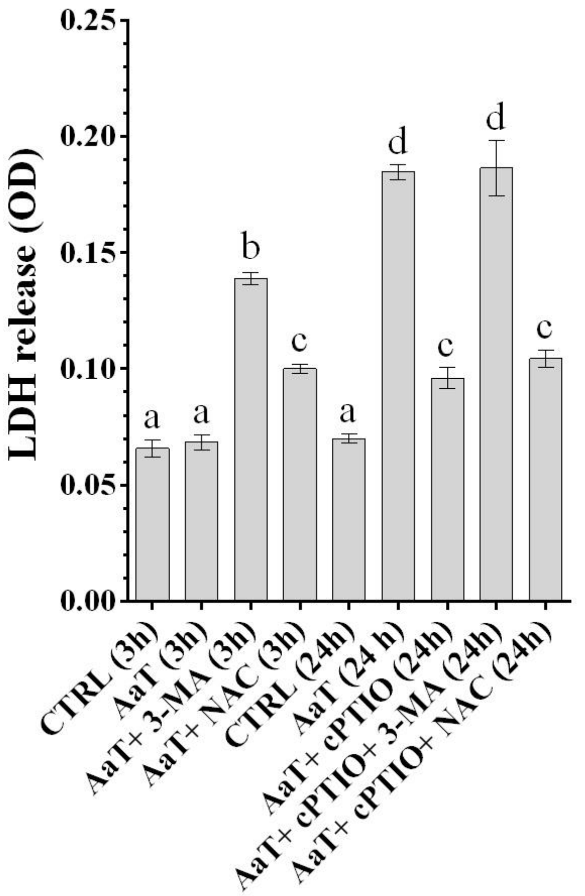

Fig. S3. The released LDH from control, AaT and AaT + inhibitors were measured from 0.5 mL of treated BY-2 cells (50 mg mL^{-1}) from each concentration, based on its ability to form coloured formazan INT. The change in absorbance was detected at 490 nm and increase in OD is directly proportionate to LDH release, i.e. necrotic cell death. Different Roman letters represent significant differences ($P<0.05$) compared to control by Holm–Sidak multiple comparison test.

Supplementary Figure S4

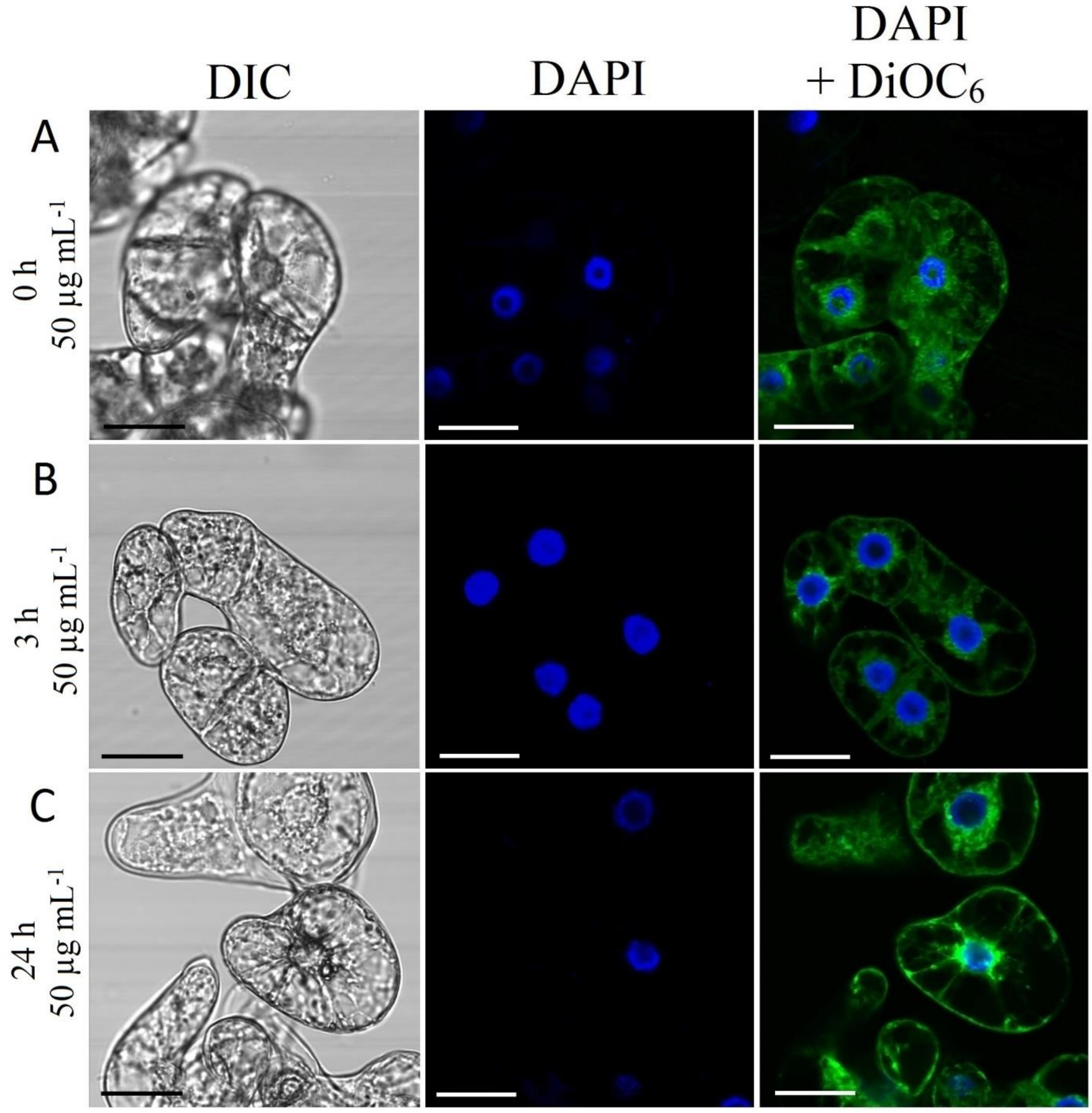

Fig. S4. Effect of AaT (50 µg mL^{-1}) on Golgi apparatus of BY-2 cells. The toxin instigated dynamics of golgi complex was assessed using lipophilic dye 3, 3'-Dihexyloxacarbocyanine iodide ($DiOC_6$). Treated cells washed in fresh medium were fixed with 3.7 % paraformaldehyde, and were stained with 1 µg mL^{-1} 4′, 6-Diamidine-2′-phenylindole dihydrochloride (DAPI) followed by 10 µM of $DiOC_6$ for 30 min at RT[3]. After thorough washing, cells were observed

under Olympus IX81 (Olympus, Singapore) confocal microscope. Olympus FLUOVIEW Ver. 04.02.02.09 (Olympus, Singapore) software was used to acquire the fluorescent images. (A) Normal Golgi distribution at 0 h. (B) Onset of peri-nuclear Golgi distribution after 3 h. (C) Peri-nuclear Golgi distribution after 24 h. Scale bars denote 20 μm. This trend was consistently observed in multiple fields in different independent experiments.

References:

1. Babula, P., Adam, V., Kizek, R., Sladký, Z. & Havel, L. Naphthoquinones as allelochemical triggers of programmed cell death. *Environ*. *Exp*. *Bot*. **65**, 330–337 (2009).

2. Towill, L. E. & Mazur, P. Studies on the reduction of 2, 3, 5-triphenyltetrazolium chloride as a viability assay for plant tissue cultures. *Can. J. Botany* **53**, 1097–1102 (1975).

3. Kawazu, T., Kawano, S. & Kuroiwa, T. Distribution of the Golgi apparatus in the mitosis of cultured tobacco cells as revealed by $DiOC_6$ fluorescence microscopy. *Protoplasma* **186**, 183–192 (1995).
